# Resting coronary flow drives the daily pattern in coronary flow reserve in patients with chest pain without obstructive epicardial stenosis

**DOI:** 10.3389/fcvm.2023.1057692

**Published:** 2023-01-25

**Authors:** Saurabh S. Thosar, Sahar Taqui, Brian Davidson, Todd Belcik, James Hodovan, Sean P. M. Rice, Jonathan R. Lindner

**Affiliations:** ^1^Oregon Institute of Occupational Health Sciences, Portland, OR, United States; ^2^OHSU Knight Cardiovascular Institute, School of Medicine, Portland, OR, United States; ^3^OHSU School of Nursing, Portland, OR, United States; ^4^OHSU-PSU School of Public Health, Oregon Health & Science University, Portland, OR, United States; ^5^Division of Cardiovascular Medicine, Robert M. Berne Cardiovascular Research Center, Charlottesville, VA, United States

**Keywords:** myocardial contrast echocardiography, perfusion imaging, microvascular dysfunction, diurnal variation, ischemia and non-obstructive coronary artery disease, coronary flow reserve, regadenoson stress

## Abstract

**Objectives:**

Ischemia with no obstructive coronary artery disease (INOCA) is a risk factor for major adverse cardiovascular events and is characterized by abnormal coronary microvascular tone. In patients with INOCA, adverse cardiovascular events most commonly occur in the morning compared to other times of the day and night.

**Materials and methods:**

We tested whether coronary microvascular function varies diurnally with attenuation in the morning in patients with symptomatic coronary artery disease without significant (>50%) epicardial stenosis. We evaluated data from 17 patients studied in the AM (700–1159 h) and 11 patients in the PM (1200–1800 h). Coronary microvascular function was measured using perfusion contrast imaging at rest and after infusion of intravenous regadenoson. We calculated microvascular flow reserve as the ratio of hyperemic to resting flow. Along with independent sample *t*-tests, we performed bootstrapping procedures to test mean differences between AM and PM groups, using the bias-corrected and accelerated method with 5,000 bootstrapped samples.

**Results and conclusion:**

The AM and PM groups were matched for demographic and existing risk factors. Coronary microvascular flow reserve was ∼33% higher in the AM compared to the PM (*P* = 0.025, BCa 95% CI [0.25, 1.64]; Hedge’s *g* = 0.89, 95% CI [0.11, 1.66]) as a result of significantly lower resting flow (∼50%) in the AM compared to the PM (*P* = 0.03, *M*_Diff_ = −56.65, BCa 95% CI [−118.59, −2.12]; Hedge’s *g* = −0.86, 95% CI [−1.60, −0.06]). Our observations are of clinical value and can influence diagnosis and treatment in the clinic based on the time of day of measurements.

## Introduction

Cardiovascular (CV) disease is the leading cause of mortality in the United States (US), and ischemic heart disease accounts for most of these deaths in the US (1). More than 700,000 US adults suffer from a new myocardial infarction or die due to ischemic heart disease each year (2), and nearly 10 million US adults suffer from angina, the primary symptom of ischemic heart disease (2). Patients with angina and demonstrable ischemia on a stress test are treated under the presumptive diagnosis of obstructive coronary artery disease, which has a well-established care pathway (3). Yet, in extensive population studies, it has been shown that up to 50% of patients with angina have ischemia and non-obstructive coronary artery disease (INOCA), defined as < 50% occlusion in any epicardial artery (4). INOCA significantly increases the risk of adverse CV events, including myocardial infarction and severe angina that typically cluster in the morning than other times of the day (5–7). In over 50% of INOCA patients, anginal symptoms are caused by abnormal regulation of coronary microvascular tone at rest or during stress. (8). Of the vasoactive pathways that control arteriolar tone, many have been shown to have a diurnal pattern, including sympathetic activity, plasma cortisol, and endothelial-dependent vasodilation (9). Yet, little is known regarding whether coronary microvascular function varies with a similar daily pattern in patients with INOCA. This issue is also relevant for patients with obstructive coronary artery disease since microvascular dysfunction can increase symptoms through loss of compensatory autoregulation (10). This work aimed to test if coronary microvascular function has a diurnal variation. We hypothesized that coronary microvascular function measured as coronary microvascular flow reserve would be attenuated in the morning compared to the afternoon.

## Methods

**FIGURE 1:**
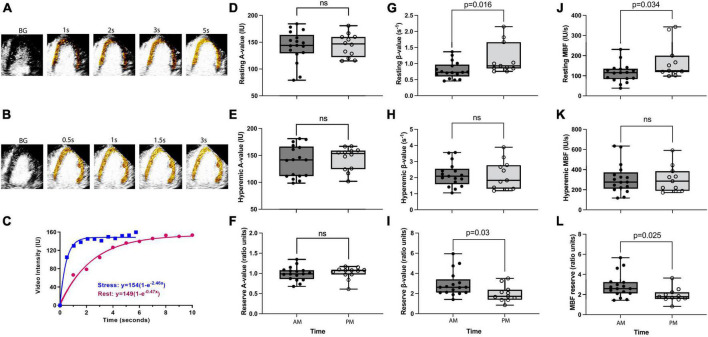
Diurnal variation in coronary microvascular function. Panels **(A,B)** are examples of background (BG) subtracted color-coded myocardial contrast echocardiographic images from the apical four-chamber view during rest and stress, respectively, after the destructive pulse sequence illustrating replenishment. Panel **(C)** is an example of time-intensity data from the myocardium at rest and during regadenoson stress. During stress, the video intensity plateaus between 1 and 2 s and with a severalfold increase in the β (microvascular flux rate) value. Panels **(D–F)** show resting, hyperemic, and reserve A values (i.e., microvascular capillary volume), respectively. There is no difference between AM and PM groups for either of these parameters. Panels **(G–I)** show resting, hyperemic, and reserve β values, respectively. A significantly lower resting β value in the AM group leads to a significantly increased β reserve in the AM, whereas hyperemic β is not different between groups. Panels **(J–L)** show resting, hyperemic, and reserve myocardial blood flow [MBF (A × β)] values, respectively. Significantly lower resting MBF in the AM group leads to a significantly increased MBF reserve in the AM, whereas hyperemic MBF is not different between groups. *P*-values stated where *P* < 0.05, ns refers to a *P*-value of >0.05.

All procedures followed were following the ethical standards of the responsible committee on human experimentation and with the Declaration of Helsinki of 1975, as revised in 2000. The study was approved by the Oregon Health & Science University Institutional Review Board. Informed consent was obtained from all patients included in the study. A comprehensive description of methods has been previously described (11). Briefly, volunteers (*n* = 28) with suspected symptomatic coronary artery disease who were referred for computed tomographic angiography but who had no significant (>50%) coronary stenosis were recruited. Myocardial contrast echocardiography quantitative perfusion imaging (iE33; Philips Ultrasound, Andover, MA, USA) was performed during a continuous infusion (0.075–0.10 ml/min) of lipid-stabilized octafluoropropane microbubbles (Definity; Lantheus Medical Imaging, North Billerica, MA, USA). Power modulation imaging at a mechanical index of 0.12–0.14 was performed in the apical views. End-systolic frames were acquired for ten seconds after a high-power frame sequence which was applied to null microbubble signal in the imaging sector. Images were acquired at rest and during vasodilator stress produced by intravenous administration of regadenoson (0.4 mg). For quantitative analysis of perfusion, time-intensity curves were modeled to calculate microvascular flux rate and functional microvascular blood volume, the product of which represents microvascular blood flow (Figures 1A–C). The analysis was conducted in a time-blinded fashion. Data were divided into a morning (AM) group (700–1159 h) and an evening (PM) group (1200–1800 h). Echocardiographic data were also collected for stroke volume, cardiac output, stroke work, and cardiac work at rest. Means were compared between the AM and PM groups using independent sample *t*-tests, with an alpha level of 0.05. First, known correlates with coronary microvascular flow reserve (e.g., age, BMI, and blood pressure) were compared. Significantly different variables were identified for possible inclusion as covariates in the main analysis. In order to account for the small sample size, Cohen’s *d* with Hedge’s *g* correction were computed with a 95% confidence interval for main outcomes. Additionally, bootstrapping procedures were applied to mean differences, using the bias-corrected and accelerated method with 5,000 bootstrapped samples. The resulting 95% confidence intervals around the mean differences were inspected; if 0 was not included in the interval, then the difference can be interpreted as statistically significant. Bootstrapping was used as a robust method to account for small sample sizes (i.e., bootstrapped estimates with samples ≥10 are reliable) (12) and potential issues with normality (13).

## Results

Eighteen women and 10 men were studied (age: 48 ± SD 6 years). Risk factors included hypertension (*n* = 7), dyslipidemia (*n* = 10), diabetes mellitus (*n* = 2), and smoking (*n* = 4). There were no significant differences in the AM (*n* = 17) versus PM (*n* = 11) group for age, body mass index, the prevalence of risk factors, or the hemodynamic and myocardial work determinants of oxygen requirement and perfusion (Table 1). On myocardial contrast echocardiography perfusion imaging, microvascular flow reserve calculated as the ratio of hyperemic to resting perfusion was significantly higher (*M*_Diff_ = 0.95, BCa 95% CI [0.25, 1.64]) in the AM than in the PM group (Hedge’s *g* = 0.89, 95% CI [0.11, 1.66], Figure 1L). This difference was significant even after controlling for systolic blood pressure (*M*_Diff_ = 0.74, BCa 95% CI [0.09, 1.39]), heart rate (*M*_Diff_ = 0.86, BCa 95% CI [0.09, 1.62]), and rate pressure product (*M*_Diff_ = 0.68, BCa 95% CI [0.11, 1.59]), respectively. Furthermore, the group difference in microvascular flow reserve occurred not because of differences in hyperemic myocardial blood flow (*M*_Diff_ = 6.21, BCa 95% CI [−98.79, 110.86], Figure 1K), but rather because of lower resting myocardial blood flow in the AM than in PM (*M*_Diff_ = −56.65, BCa 95% CI [−118.59, −2.12]; Hedge’s *g* = −0.86, 95% CI [−1.60, −0.06], Figure 1J), which could not be explained by increased myocardial work. Additionally, the diurnal differences in resting myocardial blood flow were entirely due to resting microvascular flux rate and not microvascular blood volume (Figures 1D–I). See Table 2 for detailed comparisons of parameters. Even though myocardial work was not different between groups, we further compared resting myocardial blood flow and resting beta normalized to cardiac work. We found that resting myocardial blood flow normalized to cardiac work was 50% lower in the AM (0.0002937 ± 0.000047 IU/ml × mmHg × bpm) compared to PM (0.0004467 ± 0.000010), but this was not significantly different (*M*_Diff_ = 0.000001, BCa 95% CI [−0.00000002, 0.000002]; Hedge’s *g* = 0.64, 95% CI [−0.12, 1.39]). Likewise, the resting microvascular flux rate normalized to myocardial work was also 45% lower in the AM (0.00000196 ± 0.000000248/ml × mmHg × bpm × s) compared to the PM (0.00000285 ± 0.000000604), but this too was not statistically different (*M*_Diff_ = 0.00015, BCa 95% CI [−0.00005, 0.00037]; Hedge’s *g* = 0.57, 95% CI [−0.19, 1.31]).

**TABLE 1:** Demographics and cardiovascular indices between AM and PM groups.

Variable	AM group (*N* = 17) Mean ± SD	PM group (*N* = 11) Mean ± SD	*t*-Value	*P*-value	Bootstrapped bias corrected and accelerated 95% CI
Age (years)	48.0 ± 6.1	47.5 ± 6.9	0.19	0.85	−4.8, 5.7
Body mass index (kg/m^2^)	27.7 ± 5.4	30.7 ± 8.6	−1.04	0.32	−8.4, 2.4
Heart rate (bpm)	69 ± 11	74 ± 15	−1.03	0.31	−16, 5
Systolic blood pressure (mmHg)	113 ± 15	126 ± 20	−1.90	0.07	−27, 1
Diastolic blood pressure (mmHg)	68 ± 10	72 ± 16	−0.90	0.38	−16, 6
Mean arterial pressure (MAP)	83 ± 10	90 ± 17	−1.39	0.18	−3.6, 18.3
Stroke volume (ml)	77.0 ± 13.8	73.9 ± 12.6	0.56	0.58	−7.8, 14.8
Cardiac output (L/min)	5.4 ± 1.3	5.2 ± 0.8	0.39	0.70	−0.6, 1.0
Stroke work (ml × mmHg)	6,427 ± 1,489	6,673 ± 1,619	−0.39	0.71	−1,395, 941
Cardiac work (ml × mmHg × bpm)	443,995 ± 135,883	468,881 ± 160,864	−0.41	0.70	−140,080, 84,889
Rate pressure product (mmHg × beats/min)	7,866 ± 1,847	9,481 ± 2,909	1.64	0.12	−3,602, 340
Count of high risk on CT-A	4	3			
Count of low-risk on CT-A	13	8			
Risk factors (counts)	3 hypertension	4 hypertension			
	1 diabetes	1 diabetes			
	4 hyperlipidemia	3 hyperlipidemia			
	1 low HDL	1 low HDL			
	1 hypertriglyceridemia				

*t*-Values are from independent sample *t*-tests.

**TABLE 2:** Parametric data comparisons.

Variable	AM group (*N* = 17) Mean ± SD	PM group (*N* = 11) Mean ± SD	*t*-Value	*P*-value	Hedge’s *g* (95% CI)	Bootstrapped bias corrected and accelerated 95% CI
Resting A value	142.1 ± 29.5	145.1 ± 22.2	−0.29	0.78	−0.11 (−0.84, 0.63)	−22.6, 17.3
Hyperemic A value	138.8 ± 29.3	143.2 ± 22.2	−0.43	0.67	−0.16 (−0.90, 0.58	−22.7, 15.5
Reserve A value	1.0 ± 0.2	1.0 ± 0.2	−0.09	0.93	−0.04 (−0.77, 0.70)	−0.1, 0.1
Resting β value	0.8 ± 0.3	1.2 ± 0.5	−2.27	0.04	−0.96 (−1.74, −0.18)	−0.7, −0.1
Hyperemic β value	2.2 ± 0.7	2.2 ± 0.9	0.06	0.95	0.02 (−0.72, 0.76)	−0.7, 0.7
Reserve β value	2.9 ± 1.2	2.0 ± 0.8	2.25	0.03	0.85 (0.07, 1.61)	0.2, 1.7
Resting coronary flow	114.0 ± 47.6	170.7 ± 87.2	−2.23	0.04	−0.84 (−1.60, −0.06)	−118.6, −2.1
Hyperemic coronary flow	310.6 ± 158.5	304.4 ± 130.0	0.11	0.91	0.04 (−0.69, 0.78)	−98.8, 110.9
Coronary microvascular flow reserve	2.9 ± 1.2	1.9 ± 0.7	2.37	0.03	0.89 (0.11, 1.66)	0.3, 1.6

## Discussion

In patients with chest pain without epicardial stenosis, there is an increased morning prevalence of adverse cardiovascular events, including myocardial infarction. These events are possibly related to impaired physiological responses to morning-specific activities, including increased physical or mental stress (14). Because coronary microvascular flow reserve is attenuated in these patients, we expected it to be preferentially lower in the AM compared to PM to coincide with the timing of adverse cardiovascular events (15). Surprisingly, we found that the myocardial blood flow reserve was approximately 33% higher in the AM compared to the PM group. This increased myocardial blood flow reserve in the AM group was a direct consequence of about 50% lower resting myocardial blood flow even though the AM and PM groups’ myocardial demand (e.g., cardiac work) was not different. Parametric analysis showed that these changes were not attributable to functional microvascular blood volume but rather microvascular flux rate. Even after normalization to cardiac work, resting myocardial blood flow and resting microvascular flux rate remained ∼50% lower in the AM compared to the PM. Although these normalized differences were not statistically significant, they are likely clinically meaningful. Our results are similar to those reported by Fujita and Franklin, who discovered that in conscious dogs, resting coronary flow was significantly lower in the morning than in the evening (16). Changes in oxygen extraction may compensate for differences in lower resting blood flow, but under pathological conditions (e.g., vasospastic angina or microvascular spasm), these patients would be susceptible to myocardial damage due to lack of blood flow (17). Indeed, in patients with non-obstructive coronary artery disease, vasospastic angina is common due to both anatomical and physiological changes in the coronary microvasculature (18, 19). Furthermore, coronary microvascular dysfunction, including attenuated responses to vasodilators and exaggerated sensitivity to vasoconstrictors, is mechanistically linked to vasospastic angina (20, 21). Our results point to two putative mechanisms. A possible time-of-day effect on myocardial metabolism, as seen in rodents, could lead to changes in resting myocardial blood flow between AM and PM (22). Secondly, a morning increase in α-1 sympathetic activity could explain the decrease in resting myocardial blood flow (23).

Previously, Toyoda et al. colleagues have demonstrated that in a small sample of healthy men (*n* = 15), coronary flow velocity reserve measured using transthoracic Doppler echocardiography is significantly lower in the morning (7 AM) compared to other times of the day (24). Conversely, Fukuda et al. studied 20 young, healthy men and showed that coronary flow velocity increased across the morning from 7 AM to 11 AM but then decreased at 9 PM (25). The differing results between our and previous investigations could be due to subject demographic variability or the timing of measurements. For instance, our sample consisted of 18 midlife female and 10 midlife male participants, whereas previous reports are limited to young, healthy men. Our measurements are divided by AM versus PM, whereas earlier reports had fixed time points, with unclear discussion on whether the first measurement affected the subsequent measurements. Therefore, our study adds clear value to this field of research because our midlife participants had non-obstructive coronary artery disease. Furthermore, our results emphasize the importance of carefully considering resting myocardial blood flow and normalizing it to cardiac work before making important treatment decisions (18).

This retrospective analysis is entirely unrelated to the aims of the previously published primary data [(11), clinical trial registration NCT02465554]. Nonetheless, the relatively low sample size and the lack of a repeated measures design and non-randomization are drawbacks. Subjects studied at different times did not have an unequal distribution of risk factors. However, we did not measure variables that could affect coronary blood flow, including hemoglobin or renal function parameters (26, 27). Even though we conducted bootstrapping to overcome sample size limitations, these results need to be replicated in a properly powered prospective study. Similarly, future studies should control for hemoglobin levels, renal function parameters, and the systemic disease status of patients (26–28). Notwithstanding the limitations, our analyses were time-blinded, and the resultant effect sizes (Hedge’s *g*) for the differences between the AM and PM groups are large. These data on diurnal variations in resting and reserve myocardial blood flow in patients with suspected INOCA are a first and should be considered hypothesis-generating for their potential impact on clinical practice. Indeed, diagnosis and treatment decisions in the clinic are not necessarily based on a measurement’s time of day.

## Conclusion

In a retrospective study, we show a diurnal variation in myocardial blood flow reserve in patients with suspected INOCA, almost entirely as a result of microvascular flux rate-dependent attenuation of resting morning myocardial blood flow. Our results generate important future questions. These results need to be confirmed in a prospective study, especially considering the magnitude of the differences. Furthermore, whether the observed daily changes are due to endogenous circadian rhythms in coronary microvascular physiology needs to be further investigated.

## Data availability statement

The original contributions presented in this study are included in the article/supplementary material, further inquiries can be directed to thosar@ohsu.edu.

## Ethics statement

The studies involving human participants were reviewed and approved by the Oregon Health & Science University Institutional Review Board. The patients/participants provided their written informed consent to participate in this study.

## Author contributions

JL obtained the funding, supervised the study, and collected the data. STa, BD, and JL contributed to the study design. TB and JH contributed to the data collection and analysis. SST contributed to the data analysis and interpretation and drafted the manuscript. SR substantially contributed to the statistical analysis and data interpretation. All authors contributed to the intellectual components of the manuscript and agreed with its contents.
